# The expansion of later Acheulean hominins into the Arabian Peninsula

**DOI:** 10.1038/s41598-018-35242-5

**Published:** 2018-11-29

**Authors:** Eleanor M. L. Scerri, Ceri Shipton, Laine Clark-Balzan, Marine Frouin, Jean-Luc Schwenninger, Huw S. Groucutt, Paul S. Breeze, Ash Parton, James Blinkhorn, Nick A. Drake, Richard Jennings, Patrick Cuthbertson, Abdulaziz Al Omari, Abdullah M. Alsharekh, Michael D. Petraglia

**Affiliations:** 1 Department of Archaeology, Max Planck Institute for the Science of Human History, Jena, Germany; 2 Research Laboratory for Archaeology and the History of Art, School of Archaeology, University of Oxford, 36 Beaumont Street, Oxford, OX1 2PG UK; 3 Centre of Excellence for Australian Biodiversity and Heritage, Australian National University, Canberra, Australia; 4 British Institute in Eastern Africa, Nairobi, Kenya; 5 Department of Geosciences, Freiburg, Germany; 6 Department of Geography, Kings College London, London, UK; 7 Human Origins and Palaeoenvironments Research Group, Department of Social Sciences, Oxford Brookes University, Headington Campus, Gipsy Lane, Oxford, OX3 0BP UK; 8 Mansfield College, University of Oxford, Oxford, OX1 3TF UK; 9 Department of Geography Royal Holloway, University of London, Egham, Surrey, TW20 0EX UK; 10 School of Natural Sciences and Psychology, Liverpool John Moores University, James Parsons Building, Byrom Street, Liverpool, L3 3AF UK; 11 Saudi Commission for Tourism and National Heritage, Riyadh, Saudi Arabia; 12 Department of Archaeology, College of Archaeology and Tourism, King Saud University, Riyadh, Saudi Arabia

## Abstract

The Acheulean is the longest lasting cultural–technological tradition in human evolutionary history. However, considerable gaps remain in understanding the chronology and geographical distribution of Acheulean hominins. We present the first chronometrically dated Acheulean site from the Arabian Peninsula, a vast and poorly known region that forms more than half of Southwest Asia. Results show that Acheulean hominin occupation expanded along hydrological networks into the heart of Arabia from Marine Isotope Stage (MIS) 7 until at least ~190 ka – the youngest documented Acheulean in Southwest Asia. The site of Saffaqah features Acheulean technology, characterized by large flakes, handaxes and cleavers, similar to Acheulean assemblages in Africa. These findings reveal a climatically-mediated later Acheulean expansion into a poorly known region, amplifying the documented diversity of Middle Pleistocene hominin behaviour across the Old World and elaborating the terminal archaic landscape encountered by our species as they dispersed out of Africa.

## Introduction

The Acheulean represents a key stage in hominin evolution, characterized by the production of large cutting tools such as handaxes for over ~1.5 million years^1–5^. Given the rarity of hominin fossils, mapping the chronological and geographic spread of the Acheulean is critical for reconstructing patterns of hominin expansion and evolution in different regions of the Old World. It is also crucial for defining the terminal Acheulean landscape encountered by hominins using Middle Palaeolithic technology, including *Homo sapiens*. However, considerable gaps remain in our understanding of the spatial and temporal distribution of the Acheulean.

**Figure 1:**
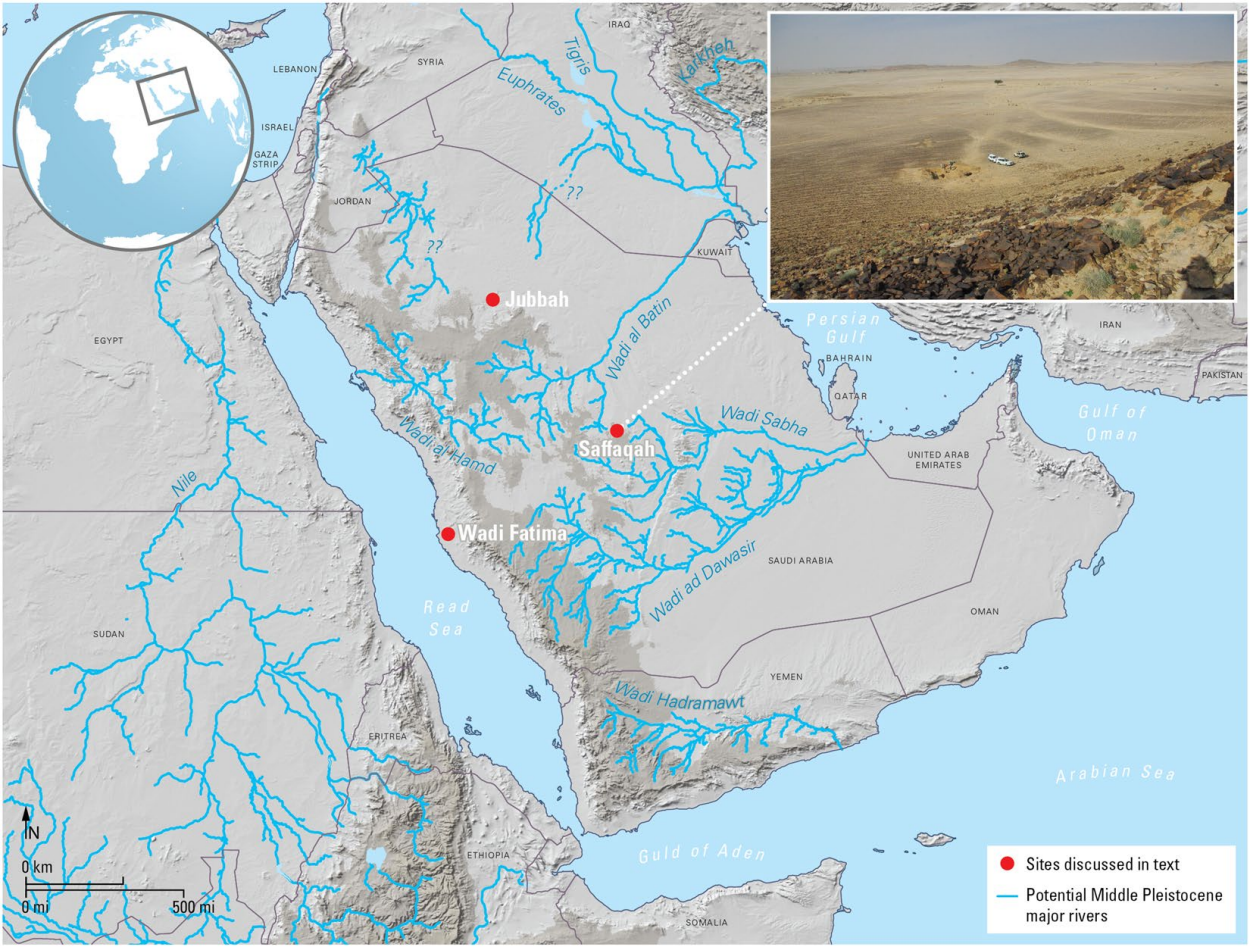
Map showing locations of major river systems and Arabian sites noted in text (**a**); view of surrounding plain from the top of the andesite dyke: Trench 1 excavations are to the left of the jeeps (**b**).

Currently, little is known about the Acheulean in the Arabian Peninsula, a critical region situated at the crossroads between Africa and Eurasia. Covering 3.2 million km^2^, the Arabian Peninsula has long been incorporated into narratives of early Eurasian colonization^6^ and numerous Acheulean sites with shared technological characteristics have been documented^7–10^. However, data required to refine and develop Acheulean dispersal hypotheses has been limited by the fact that the vast majority of documented sites are deflated surface sites lacking stratigraphy and chronometric age estimates. The only known stratified Acheulean site is Saffaqah, situated in the Dawadmi region of the Nejd plateau (Fig. 1). Saffaqah was first identified and excavated by Norman Whalen and colleagues in the 1980s^11^. Their 33 m^2^ excavation (Trench 1) resulted in the recovery of 8,395 buried artefacts. However, Whalen and colleagues^11^ did not discuss the stratigraphy nor artefact distributions within the excavations in any detail. They observed calcrete in Trench 1 from 30 cm downwards, while sterile sediment was reached at depths of 1.33–1.49 m, and degrading granite bedrock at 1.62 m. Uranium-thorium ages on calcrete adhering to the artefacts suggested that the deposits were at least 200,000 years old, but the ages could not be considered secure (see Methods) and were not further refined.

Given the importance of Saffaqah, a re-assessment of the site’s stratigraphy, archaeology and chronology was conducted by the Palaeodeserts Project in 2014 through re-excavation and extension of Trench 1, and a study of the surrounding landscape^10^. As reported here, new field investigations now also provide the first secure dates for what is still the only known stratified Acheulean site in the Arabian Peninsula.

## Results

The Dawadmi region of central Saudi Arabia is characterized by a flat plain of Proterozoic igneous bedrock intruded by younger felsic and mafic dykes, and overlain in places by Quaternary aeolian and fluvial sediments. The climate is currently arid, although an intensification of the African Summer Monsoon and its incursion into Arabia brought increased summer rainfall to the Peninsula in the past^12,13^. Palaeoenvironmental records and climate model simulations indicate that increased humidity occurred during insolation maxima, in particular during interglacials and interstadials, such as those during Marine Isotope Stage 7 (MIS 7, ~240–190 ka) and the interstadials of MIS 5 (~130–75 ka)^12,14^. During these periods, the landscape of Arabia featured extensive river networks, lakes and wetlands with widespread vegetative increases. The Saffaqah site itself is situated near the Central Saudi Arabian town of Dawadmi. It is located beside the most prominent andesite dyke in the region, which rises ~60 m above the surrounding plain and is located on the northern flank of the dyke, below its highest point (Fig. 1). Our systematic survey revealed that Saffaqah is the largest Acheulean site yet documented in Arabia and is surrounded by a dense Acheulean landscape^10,15^.

Palaeohydrological reconstruction demonstrated that the site sits at the interface between two major extinct river systems: the Wadi al Batin and the Wadi Sahba, major riparian corridors that flowed into the northern and southern ends of the Gulf, respectively (Fig. 1)^15,16^.

**Figure 2:**
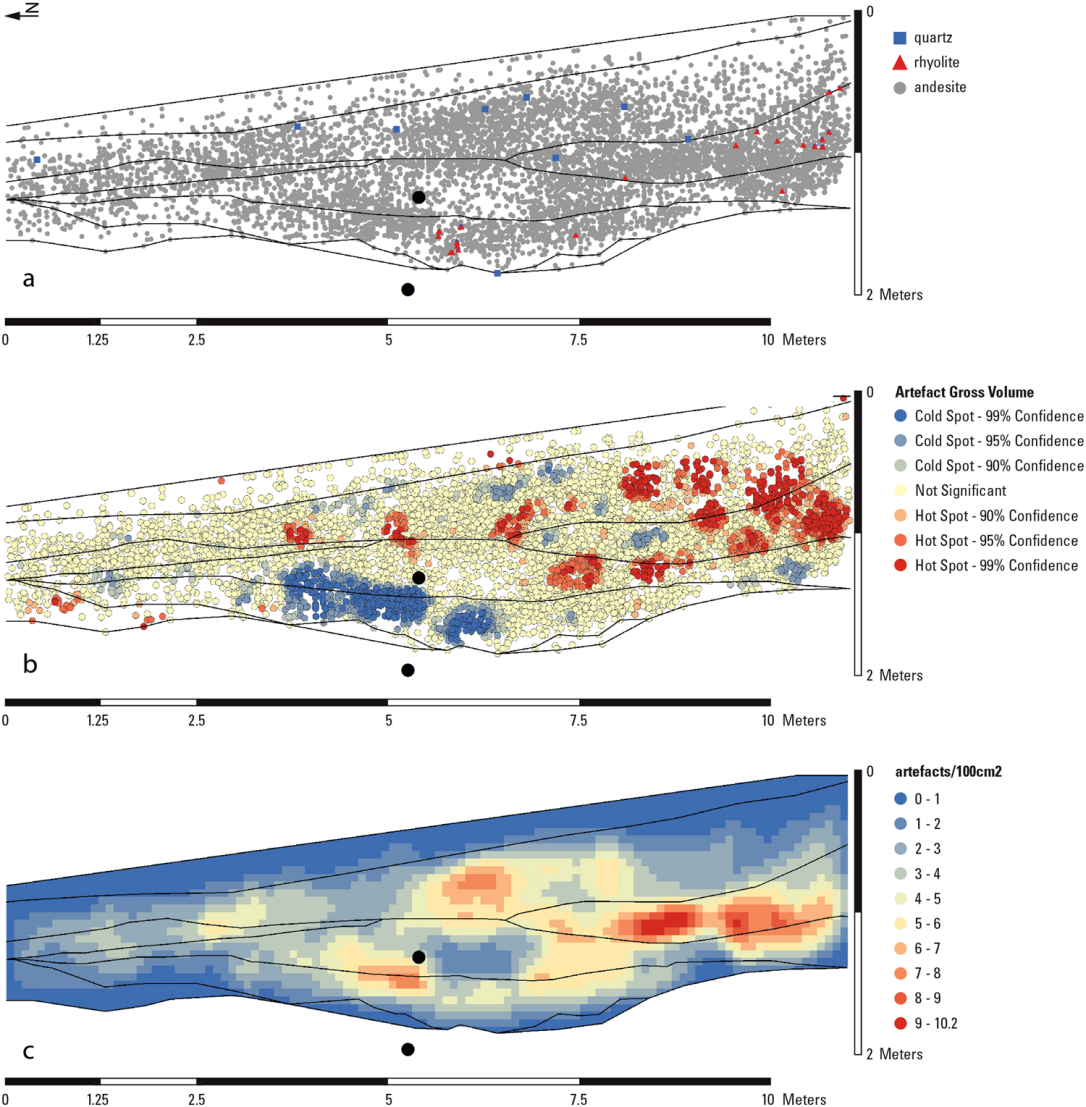
Analysis of the artefact distributions within Trench 1 combining Whalen’s^11^ 3D artefact co-ordinates with the newly recorded stratigraphy showing (**a**) distribution of artefacts split by raw material types. (**b**) Hot-Spot analysis of gross artefact volume (L × W × T) indicating concentrations of significantly high and low artefact volumes; (**c**) total artefact densities. Filled black circles represent the locations of sediment samples for luminescence dating. Modified from Shipton and colleagues^11^.

**Figure 3:**
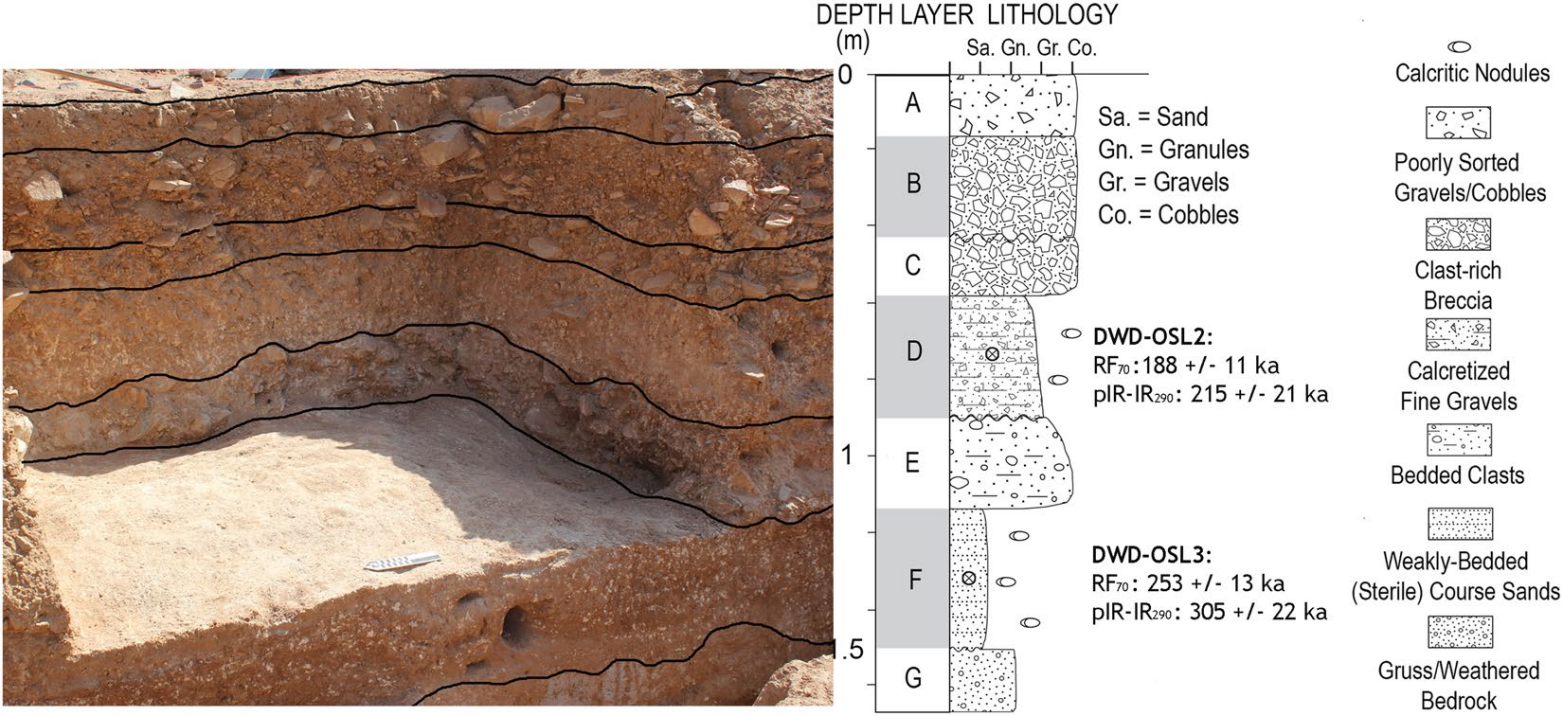
Stratigraphy of Saffaqah, determined from new excavations with sediment samples for luminescence dating bracketing Layer E. Modified from Shipton and colleagues^11^.

We excavated several test trenches running north downslope from Whalen and colleagues’ Trench 1 that confirmed increasingly attenuated stratigraphic sequences lacking buried artefacts. Re-excavation of Trench 1 itself revealed a shallow depression holding *in situ* material, which has infilled to give a thicker stratigraphic sequence and limited post-depositional artefact movement. This depression and its infilling is also clearly visible in piece-plots of the artefacts from Whalen’s excavation (Fig. 2). We therefore extended this central part of Trench 1 by 2.5 × 1.5 m (Fig. 3).

**Figure 4:**
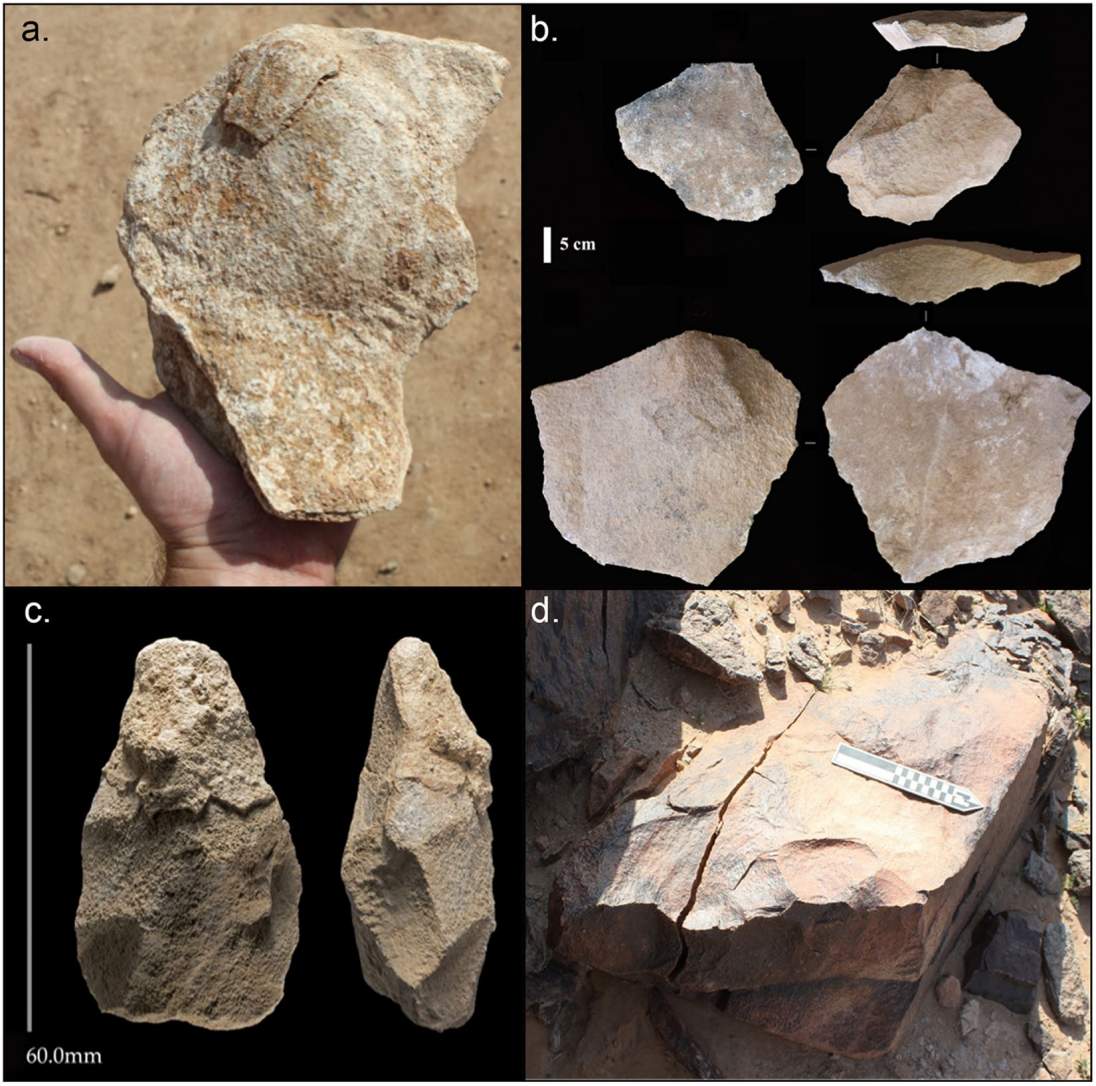
Composite figure of lithics from Saffaqah Layer E, (**a**) large flake with eraillure flake still attached; (**b**) large flakes; (**c**) typical handaxe; (**d**) giant andesite core. Modified from Shipton and colleagues^11^.

Our excavation revealed 1.53 m of sediments within which we identified seven stratigraphic units (A to G, Fig. 3). Both Layer G and the overlying Layer F are archaeologically sterile. A high density accumulation of artefacts in fresh condition, including relatively small pieces, are found concentrated within Layer E, the earliest hominin occupation of the site, matching comparable evidence from Whalen’s excavation (Figs 2 and 3). Layer E predominately comprises andesite artefacts but also included the rare exploitation of rhyolite (Fig. 2). One of the large flakes from Layer E, which was resting on the core from which it had been struck, still had its eraillure flake adhering to it (Fig. 4a) indicating that the artefacts appear to be in effectively the same positions in which they were discarded by hominins.

This initial occupation was buried by finer sediment with lower artefact densities (Layer D). The overlying deposit (Layer C) is the first of three colluvial horizons that includes a distinct concentration of both large and small artefacts, as well a second concentration of rhyolite artefacts. Lower artefact densities are observed in Layer B, which contains discrete clusters of larger artefacts and dispersed evidence for quartz exploitation.

**Figure 5:**
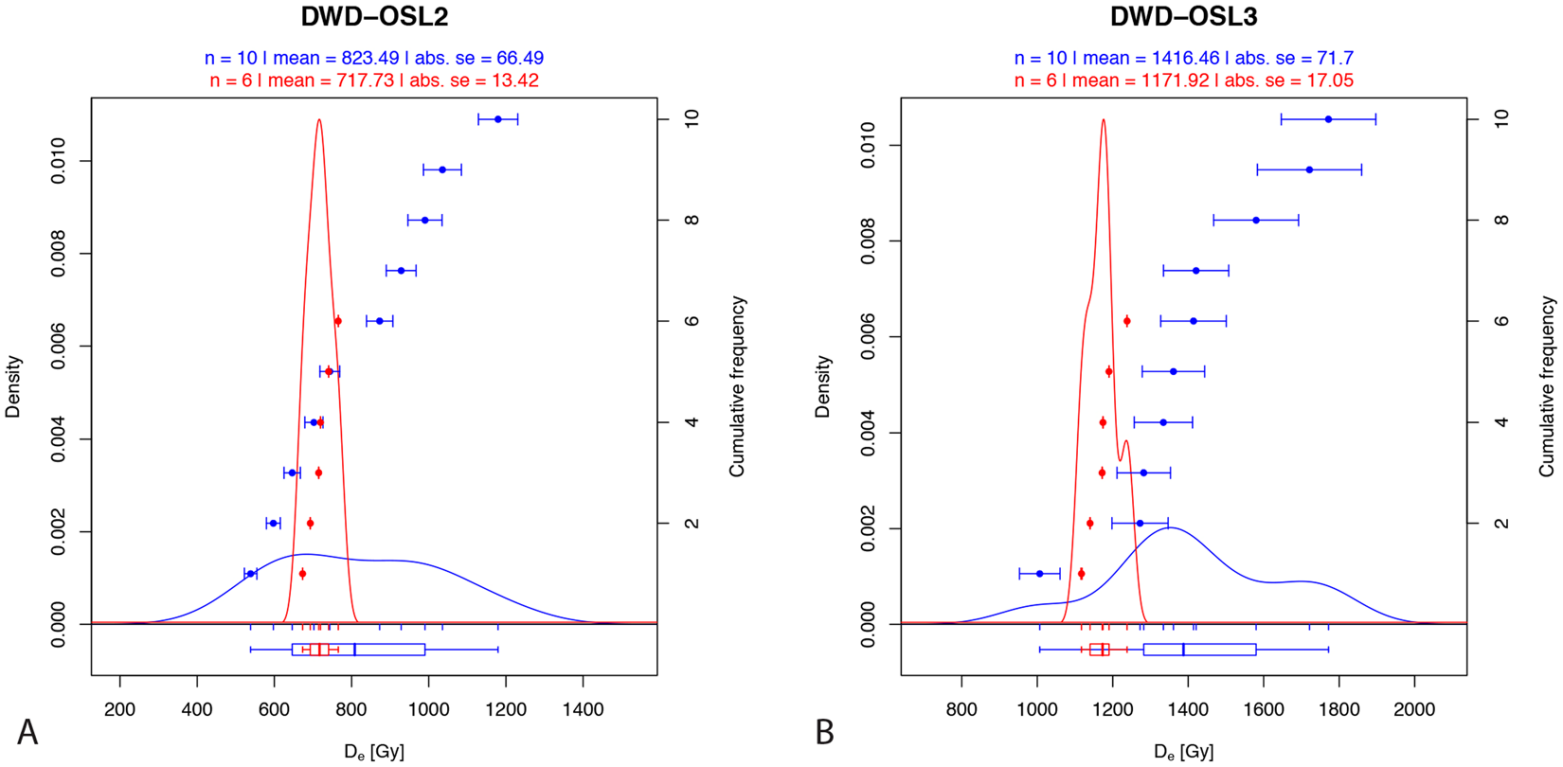
Kernel density estimate of the pIRIR_290_ (blue) and RF_70_ (red) equivalent doses (dots) and associated error bars in ascending order. The boxplot shows the distribution parameters (median as bold line, box delimited by the first and third quartile, whiskers defined by the extremes).The plot was obtained from the R (R Development Core Team, 2015) package ‘Luminescence’ version 0.8.2^60–62^.

Sediment samples were collected for luminescence dating within Layers F and D of the freshly excavated trench, two layers bracketing the basal high-density *in situ* lithics in Layer E (Figs 2, 3 and 5, Tables 1–3). Samples were analysed using the post-IR elevated temperature (290 °C) infrared protocol (pIRIR_290_)^17–19^ and the infrared-radiofluorescence protocol at controlled temperature (RF_70_)^20,21^ (See Methods). pIRIR_290_ and RF_70_ De dispersions are shown in Fig. 5 and corresponding age estimates are consistent at 2 sigma, providing an average age of ~276 ka for the sterile Layer F and ~197 ka for Layer D.

**Table 1:** Radioelement contents and dose rates from Saffaqah sediment samples.

Sample	Burial depth (m)	Radioelement content			Dose rate (Gy ka^−1^)			
		K (%)	Th (ppm)	U (ppm)	Ext gamma	Ext. beta	Ext. alpha	Total
DWD-OSL2	0.85	2.0	8.0	2.0	1.06 ± 0.05	1.90 ± 0.05	0.07 ± 0.01 *0.06* ± *0.01*	3.83 ± 0.21 *3.82* ± *0.22*
DWD-OSL3	1.45	2.2	11.1	3.0	1.57 ± 0.08	2.24 ± 0.06	0.11 ± 0.02 *0.09* ± *0.02*	4.65 ± 0.24 *4.63* ± *0.24*

*a*-value is 0.08 ± 0.02 for the pIRIR_290_ ^63^ and 0.067 ± 0.012 for RF_70_ ^59^.

Values in italic are for the RF_70_ measurements.

**Table 2:** SAR characteristic, De’s measured via the pIRIR_290_ and RF_70_ protocol and luminescence age estimates. All measured aliquots were accepted for analysis; the rejection criteria applied for the pIRIR290are discussed in the text.

Sample	Protocol	#	Recycling Ratio (μ ± 1 σ)	Zero Ratio (μ ± 1 σ)	D_e_ ± se^a^ (Gy)	OD ± se^b^ (%)	Age ± se^c^ (ka)
DWD-OSL2	pIR-IR_290_	10	1.01 ± 0.01	1.21 ± 0.18	823.5 ± 66.5	23 ± 6	215 ± 21
	RF_70_	6	—	—	717.7 ± 13.4	4 ± 1	188 ± 11
DWD-OSL3	pIR-IR_290_	10	1.01 ± 0.01	1.16 ± 0.17	1416.5 ± 71.7	9 ± 6	305 ± 22
	RF_70_	6	—	—	1171.9 ± 17.1	3 ± 1	253 ± 13

^a^Unweighted mean.

^b^Overdispersion calculated using the Central Age Model^53^.

^c^Calculated using DRAC v.1.2^64^.

**Table 3:** Whalen and colleagues’^11^ Uranium-Thorium Ages on ‘caliche rind’ from artefacts from unit U-5.

Depth in mm	Age in years USGS	Age in years McMaster
700	204,000 ± 17,000	112,000 ± 15,000
860	189,000 ± 14,000	61,000 ± 9,000
860	189,000 ± 14,000	61,000 ± 9,000

The depth of the samples suggests in unit U-5 suggest the both likely come from the equivalent of our layer D. Dates were measured by B.J. Szabo of the USGS in Denver, and by H.P. Schwarz at McMaster University, Ontario.

These dates, together with the fact that central Arabia was predominantly arid during global glacial periods, indicate that artefact deposition in the intermediary Layer E occurred at the beginning of the MIS 7 interglacial. During this time, riparian and lacustrine networks of the Arabian interior were activated following an eastward extension of the African Summer Monsoon system^15,22,23^. The sharp contact between Layers F and E, and a very diffuse contact between Layers E and D also supports the notion of a quasi-continuous phase of deposition between E and D during MIS 7 and into the beginning of MIS 6.

Our new excavations at Saffaqah resulted in the recovery of over 500 lithic artefacts in stratified context. These are described in detail in Shipton and colleagues^10^. This assemblage is dominated by large flakes (the largest measuring 310 by 285 mm) that acted as biface blanks (Fig. 4b). These flakes were detached from large blocks of andesite with considerable force (Fig. 4d). Marginally and ventrally retouched flakes, cleavers and discoidal cores were also present. Biface blanks were also produced through bifacial flaking of appropriate edges on large blocks of andesite. Despite the late Acheulean date, the handaxes are minimally flaked, long ($\bar{x}$ 16  mm, σ 38) and thick ($\bar{x}$ 5  mm, σ 15) (Fig. 4c).

The artefact positioning and stratigraphic observations indicate that the lower, chronologically constrained Layer E represents an occupational surface with *in situ* knapping, while the upper layers represent successive occupations, with some minor colluvial deposition in Layers C-B likely dating to MIS 6. Excavated material exhibited no size sorting, preserving flakes as small as 13 mm, which are not found on the surface. Our spatial plotting of artefacts from Whalen’s excavation also showed that materials were unevenly distributed throughout: a small amount of quartz artefacts occurred in the upper layers, and rhyolite artefacts occurred as distinct horizontal clusters in the middle and lower parts of the excavation (Fig. 2).

## Discussion

Our results demonstrate an Acheulean presence in the Arabian Peninsula during MIS 7, and illustrate how hominins moved into marginal areas with the onset of environmental amelioration. Saffaqah also documents the youngest yet known Acheulean in Southwest Asia^24^.

The positioning of Saffaqah at the drainage divide between both the Wadi al Batin and the Wadi Sabha palaeodrainage systems, suggests that Acheulean hominins were using fluvial networks as dispersal corridors into the Peninsula’s interior (Fig. 1)^13,16,23,25,26^. Technological similarities between Saffaqah and other undated Acheulean sites strongly suggest this dispersal may have been widespread in Arabia. For example, minimally flaked handaxes with large flake blanks and cleavers have been documented in both Wadi Fatima, and the Jubbah area (Fig. 1)^27,28^, in addition to recently reported Acheulean sites in southern Arabia^29,30^.

The Acheulean technology from Saffaqah can be contrasted to Acheulean surface assemblages found in the southwestern Nefud Desert^9^. Although undated, these assemblages document very different technological characteristics within an Acheulean tradition. The southwestern Nefud assemblages lack cleavers and any large flake component, despite the apparent availability of large blocks of raw material, and feature handaxes that are highly refined, morphologically pointed and shaped from raw material blocks (*façonnage*). This constellation of technological features is similar to the Late Acheulean in the Levant^31^.

In contrast, the large flakes and cleavers of Saffaqah differ from the pene-contemporaneous Acheuleo-Yabrudian technocomplex of the Levant^10^ and are instead features more typical of the African Acheulean^32,33^. Late Acheulean sites such as Mieso (Ethiopia) are of a similar age to Saffaqah as well as manifesting large flakes and cleavers^34,35^, although it lacks the fine marginal trimming on its bifaces seen at Mieso. It therefore seems possible that Saffaqah reflects a hominin dispersal from the Horn of Africa following the northeastward migration of the African Summer Monsoon during MIS 7^34,35^. The similarities in the lithic typologies at Saffaqah across all layers spanning MIS 7 and very probably MIS 6 in the case of layers C-B, are also indicative of cultural continuity at least within the general area of the site. Light colluvial activity within Layers C-B plausibly reflects insolation peaks within MIS 6 (i.e. at ~170 and/or 155 ka), providing climate mechanisms to support a sustained hominin presence in the region during generally arid conditions. The results presented here therefore both extend the known spatial and temporal distribution of Acheulean hominins and document variation in behaviour across an expanded range and timeframe. While the age of the Acheulean sites in other parts of Arabia is not yet confirmed, the techno-typological and chronological similarities between the later Acheulean in Africa and Saffaqah indicate that several waves of dispersal may have structured the Acheulean record of Eurasia^36^.

Finally, because Saffaqah also represents the youngest yet documented Acheulean in southwest Asia, further insights are provided into the persistence of the last Acheulean hominins, the youngest of which have been documented in India in MIS 6^37^. At Saffaqah, this Acheulean presence was late enough to overlap with an emerging Middle Palaeolithic, both in the Peninsula as well as in surrounding regions, adding to the spatial diversity of Middle Pleistocene hominin behaviour^22^, and reflecting the complexity and breadth of biogeographical exchange across the Eurasian gateway. The date of ~188 ± 11 ka in Layer D provides a *terminus post quem* for the artefacts in the overlying Layers B-C, suggesting that cultural overlap continued into MIS 6. The broader regional evidence, including the presence of *Homo sapiens* in association with early Middle Palaeolithic tool assemblages at Misliya Cave in the Levant during this time^38^, provides strong indications that Saffaqah formed part of the terminal archaic landscape first encountered by our species as they dispersed out of Africa. Future research should seek to clarify the spatio-temporal character of the final Acheulean and early Middle Palaeolithic/Stone Age, as well as exploring the reasons for this complex transition.

## Methods

Sediment samples for luminescence dating were collected from the freshly opened section by inserting opaque metal tubes. Preparation and analyses were carried out at the Luminescence Dating Laboratory of the Research Laboratory for Archaeology and the History of Art, School of Archaeology, at the University of Oxford under filtered laboratory lighting (low intensity LED lighting with peak emission at 594 nm). After the sample tubes were opened, the light-exposed ends were removed for dose rate determination and the interior, light shielded sediment was retained for luminescence dating. Each sediment sample was prepared in a conventional manner in order to extract sand sized (180–255 µm) potassium (K−) feldspar mineral grains. This included wet sieving, hydrochloric acid (10%) and then hydrogen peroxide (30%) digestions, followed by heavy liquid density separation using a solution of sodium polytungstate (2.58 g cm^−3^), and a final second sieving.

For the external dose rate contribution, the beta dose rates were calculated from the uranium, thorium and potassium concentrations determined from a homogenized portion (10 g) of sediment by inductively coupled mass spectrometry (ICP-MS) and inductively coupled atomic emission spectroscopy (ICP-AES) outsourced to an accredited specialist laboratory (Actlabs, Canada). Gamma dose rates were measured on site for both samples with an Inspector 1000 gamma-ray spectrometer fitted with a 2.5′ NaI probe and calibrated against the Oxford blocks^39^ using the threshold technique^40,41^. The cosmic-ray dose rates were estimated from the equations provided by Prescott and Hutton^42^ taking into account the burial depth of the samples, the sediment density and the location of the site (altitude, latitude). Water content corrections were calculated assuming a burial average of 10 ± 4%, with correction factors from Zimmerman^43^. Assuming that each grain comprises 12.5 ± 0.5% potassium and 400 ± 100 ppm rubidium-87^44,45^, with absorption factors calculated from Brennan^46^ and the rubidium dose rate from Readhead^47^, an internal feldspar dose rate of 0.91 ± 0.15 Gy. ka^−1^ was included in the dose rate calculations (Table 1).

Equivalent doses (D_e_) were measured for ten multigrain K-feldspar aliquots (1 mm diameter) from each sample using the pIRIR_290_ protocol of Thiel *et al*.^19^. Measurements were performed on a *Lexsyg Smart* luminescence reader manufactured by Freiberg Instruments^48^ and fitted with a calibrated beta source delivering 0.134 ± 0.003 Gy.s^−1^. Aliquots were stimulated with infrared LED’s emitting at 850 ± 20 nm (200 mW cm^−^²) and the 410 nm IRSL emission signal was detected with a blue detection window (Schott BG39 3 mm and AHF Brightline HC 414/46 Interference 3.5 mm).

Net pIRIR_290_ signals were calculated by subtracting a mean background (last 50 s of stimulation) from the total signal emitted over the first 2 s^49^. Equivalent doses were calculated from each aliquot using the Analyst (v. 4.11) software developed by Duller^50–52^ and meeting the following criteria: i) detectable net natural test signal greater than three sigma above the background signal; ii) test dose error greater than 20% of the calculated test dose; iii) recycling ratios >10%; iv) calculated zero-ratio >5% of L_N_/T_N_. Based on previous results from samples collected in the Nefud Desert and discussions in Thiel *et al*.^19^ and Buylaert *et al*.^53^, fading rates were considered to be negligible and ages are uncorrected. All twenty measured aliquots had measurable signals and good SAR characteristics; no aliquots were excluded after application of the rejection criteria and none were found to be in saturation. Figure S1 (see supplementary information) shows a typical feldspar pIRIR_290_ signal and corresponding growth curve. Overdispersion values^54^ were low for both samples, with a value of 23 ± 6% calculated for DWD-OSL2, and 9 ± 6% for DWD-OSL3 (Table 1). Investigation of a modern, aeolian-deposited sample has also indicated that well-bleached feldspars have negligible residual pIRIR_290_ signal (~1 Gy).

D_e_’s were also measured using infrared-radiofluorescence (IR-RF). The IR-RF signal of K-feldspar is believed to provide a promising alternative to more established luminescence dating approaches. The technique was introduced by Trautmann *et al*.^55^ and Erfurt & Krbetschek^20^ and a reinvestigation of the IR-RF signal characteristics recently led to an improved measurement protocol, which requires keeping the temperature constant at 70 °C during measurement^21,56,57^.

IR-RF measurements were carried out on a Freiberg Instruments Lexsyg Research reader^48^ equipped with a specially designed ^90^Sr/^90^Y ring-source^58^ delivering 0.057 ± 0.003 Gy.s^−1^. IR-RF signal detection was made through a Chroma D850/40 interference filter. For bleaching, we used the built-in solar simulator equipped with different LEDs: 365 nm, max. 70 mW cm^−2^; 462 nm, max. 110 mW cm^−2^; 525 nm, max. 45 mW cm^−2^; 590 nm, max. 30 mW cm^−2^; 625 nm, max. 90 mW cm^−2^; 850 nm, max. 170 mW cm^−2^. All bleaching and measurement settings followed the suggestions made by Frouin *et al*.^21^ and the D_e_ values were obtained using the function analyse IRSAR.RF from the R ‘Luminescence’ package^21,59^. Figure S2 (see supplementary information) shows the results of the RF_70_ measurements for the two samples. For both samples, the overdispersion values are less than 5%, indicating that the RF_70_ signal has been sufficiently bleached. We note that the pIRIR_290_ and RF_70_ age estimates are consistent at 2 sigma and in stratigraphic order (Table 2).

The only independent age data available for this site consists of uranium-series dating performed upon three samples of ‘caliche rind’ collected from the underside of lithics excavated by Whalen and colleagues^11^. These dates are summarized in Table 3. Little information is available concerning the methodology of this study, as it was published as a work in progress. Therefore, the large age difference obtained for subsamples from the same tool is not explicable at the moment, but may be due to varying pre-treatments upon contaminated caliche rinds^11^. Because of this, and due to the nature of the samples (post-depositional carbonate concretion), these ages must certainly be considered as minimum estimates. While not fully reliable, these calculated ages do not conflict with the current pIRIR_290_ and RF_70_ age estimates. It is also important to note that, although these values indicate that there is post-depositional enrichment of the uranium component in the sediment, the proportion of the dose rate contributed by the uranium series in this environment is quite low. Therefore, any decay chain disequilibrium is expected to have a negligible effect on the calculated ages. Interestingly Whalen and colleagues^11^ observed that this ‘caliche rind’ exclusively occurred on the underside of artefacts, suggesting that however old it is, the artefacts have not moved since deposition.

## Electronic supplementary material

Supplementary Information

## Data Availability

Luminescence data generated during the current study are available from M.F. and J.-L.S. upon reasonable request.
