# Supplementary material for: The expansion of later Acheulean hominins into the Arabian Peninsula
Source: Sci Rep. 2018 Nov 29;8:17165. doi: 10.1038/s41598-018-35242-5 (PMC6265249; doi:10.1038/s41598-018-35242-5)
Supplement: Supplementary file 1 — Supplementary Information [file 41598_2018_35242_MOESM1_ESM.pdf]

The expansion of Late Acheulean hominins into the Arabian Peninsula

Eleanor M.L. Scerri[1,2*], Ceri Shipton[3,4], Laine Clark-Balzan[5], Marine Frouin[1], Jean-Luc Schwenninger[1], , Huw S. Groucutt[1,2], Paul S. Breeze[6], Ash Parton[7,8], James Blinkhorn[1,9], Nick A. Drake[1,6], Richard Jennings[10], Patrick Cuthbertson[1], Abdulaziz Al Omari[11], Abdullah M. Alsharekh[12], Michael D. Petraglia[1*]

[1] Department of Archaeology, Max Planck Institute for the Science of Human History, Jena, Germany.

[2] Research Laboratory for Archaeology and the History of Art, School of Archaeology, University of Oxford, 36 Beaumont Street, OX1 2PG, U.K.

[3] Centre of Excellence for Australian Biodiversity and Heritage, Australian National University, Canberra, Australia.

[4] British Institute in Eastern Africa, Nairobi, Kenya.

[5] Department of Geosciences, Freiburg, Germany.

[6] Department of Geography, Kings College London, U.K.

[7] Human Origins and Palaeoenvironments Research Group, Department of Social Sciences, Oxford Brookes University, Headington Campus, Gipsy Lane, Oxford, OX3 0BP, U.K.

[8] Mansfield College, University of Oxford, OX1 3TF, U.K.

[9] Department of Archaeology, Classics and Egyptology, University of Liverpool, U.K.

[10] School of Natural Sciences and Psychology, Liverpool John Moores University, James Parsons Building, Byrom Street, Liverpool, L3 3AF, U.K.

[11] Saudi Commission for Tourism and National Heritage, Riyadh, Saudi Arabia.

[12] Department of Archaeology, College of Archaeology and Tourism, King Saud University, Riyadh, Saudi Arabia.

Corresponding authors

*eleanor.scerri@rlaha.ox.ac.uk

*petraglia@shh.mpg.de

Supplementary Information

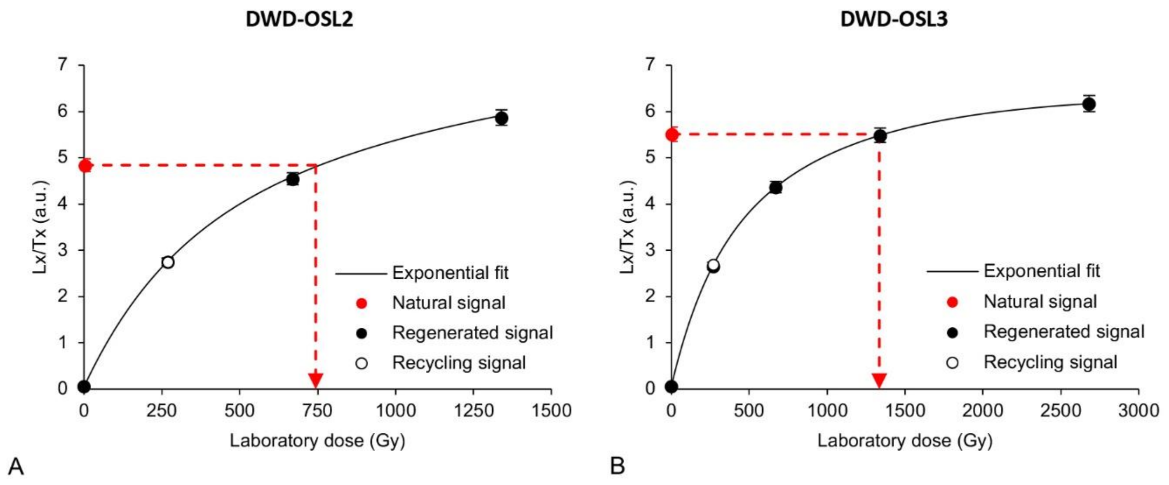

Figure S1: Typical feldspar pIRIR$_{290}$ growth curve.

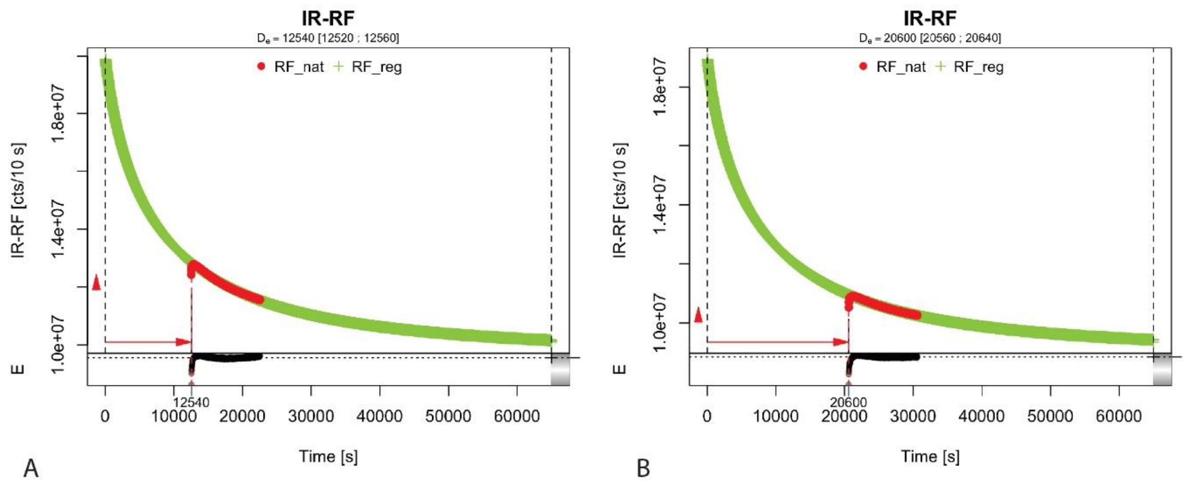

Figure S2: Results of the RF$_{70}$ measurements for the two samples.
